# Combined Immunodeficiency Evolving into Predominant CD4+ Lymphopenia Caused by Somatic Chimerism in JAK3

**DOI:** 10.1007/s10875-014-0088-2

**Published:** 2014-09-10

**Authors:** Sol A. Ban, Elisabeth Salzer, Martha M. Eibl, Angela Linder, Christoph B. Geier, Elisangela Santos-Valente, Wojciech Garncarz, Thomas Lion, Raphael Ott, Christoph Seelbach, Kaan Boztug, Hermann M. Wolf

**Affiliations:** 1 CeMM Research Center for Molecular Medicine of the Austrian Academy of Sciences, Lazarettgasse 14, AKH BT 25.3, A-1090 Vienna, Austria; 2 Immunology Outpatient Clinic, Schwarzspanierstraße 15/1, A-1090 Vienna, Austria; 3 Department of Pediatrics and Adolescent Medicine, Medical University of Vienna, Vienna, Austria; 4 Children’s Cancer Research Institute, Vienna, Austria; 5 Labdia Labordiagnostik GmbH, Vienna, Austria; 6 Kardinal Schwarzenberg‘sches Krankenhaus, Schwarzach im Pongau, Austria; 7 Research Center for Molecular Medicine of the Austrian Academy of Sciences, Lazarettgasse 14, AKH BT 25.3, A-1090 Vienna, Austria

**Keywords:** Primary immunodeficiency, idiopathic CD4+ lymphopenia, JAK3 deficiency, somatic reversion, somatic mosaicism, TCR Vβ spectratyping

## Abstract

**Purpose:**

Idiopathic CD4 lymphopenia constitutes a heterogeneous group of immunodeficiencies with characteristically low CD4+ T-cell counts with largely unknown genetic etiology. We here sought to determine the underlying molecular cause in an index family with two patients suffering from combined immunodeficiency that evolved into predominant CD4+ lymphopenia. The more severely affected index patient also presented with selective antibody deficiency against bacterial polysaccharide antigens.

**Methods:**

For the genetic analysis, we used combined homozygosity mapping and exome sequencing. Functional assays included immunoblot analysis, flow cytometry and TCR Vβ spectratyping.

**Results:**

A novel homozygous missense mutation was revealed in the kinase domain of *JAK3* (c.T3196C, p.Cys1066Arg). Further analysis showed revertant chimerism in CD8+ T-cells in both patients. The additional presence of revertant CD4+ T-cells was associated with a milder clinical and immunological phenotype in the second patient, although the role somatic chimerism plays in amelioration of disease phenotype is uncertain, as presence of revertant cells had no effect on residual CD4 cell JAK3 signaling function. Residual activity of JAK3-dependent STAT3 and STAT5 signaling was also found in immortalized B-cell lines indicating a hypomorphic nature of the described mutation which likely contributes to the milder clinical phenotype.

**Conclusions:**

We here present the first case of revertant mosaicism in JAK3 deficiency, manifesting as combined immunodeficiency evolving into predominant CD4+ lymphopenia. Revertant chimerism or hypomorphic mutations in genes typically associated with more severe T-cell deficiency should be considered when assessing patients with milder forms of combined immunodeficiencies.

**Electronic supplementary material:**

The online version of this article (doi:10.1007/s10875-014-0088-2) contains supplementary material, which is available to authorized users.

## Introduction

Idiopathic CD4 lymphopenias (ICLs) constitute an enigmatic and heterogeneous group of disorders which are collectively characterized by prolonged low CD4+ T lymphocyte count of less than 300 cells/μl or less than 20 % of lymphocytes on more than one determination in the absence of known causes such as HIV infection, malignant disease or medication [1–3]. The clinical phenotype of ICL is variable and ranges from asymptomatic laboratory abnormality of CD4+ T-cell count to increased susceptibility to infections, opportunistic infections and autoimmune diseases [3, 4]. Current knowledge on the molecular pathogenesis of ICL is limited.

Genetic studies of patients with combined immunodeficiency (CID) with predominant CD4 cell deficiency have revealed mutations in genes encoding regulatory factors of the expression of MHC class II molecules such as *CIITA*, *RFXANK*, *RFX5* or *RFXAP* [5–9]. The associated disease is termed MHC class II deficiency, characterized by low numbers of CD4+ T-cells while numbers of CD8+ T-cells are normal or elevated [10]. Furthermore, mutations in *P56LCK*, a tyrosine kinase in the downstream of the TCR activation pathway, were described to cause CID with CD4 deficiency [11]. Recently, a mutation in *MAGT1*, a gene encoding a Mg2+ transporter, was reported to cause a disorder associated with CD4 deficiency denominated as XMEN – X-linked immunodeficiency with magnesium defect and EBV infection and neoplasia [12]. Other studies have reported CID with CD4 lymphopenia in patients bearing hypomorphic mutations in genes which are typically associated with severe combined immunodeficiency (SCID) phenotype, such as *RAG1*, which is known to cause SCID when mutated in amorphic manner [13].

In this study, we investigated a consanguineous family with two affected siblings suffering from CID that evolved into predominant CD4 lymphopenia in order to define hitherto unknown genetic etiologies underlying this condition.

## Methods

### Patients

The protocol for this study was approved by the Ethics Committee at the Medical University of Vienna, Austria. Blood samples from index patients and their family members from a Turkish family were obtained with informed consent in agreement with the Declaration of Helsinki.

### DNA Isolation

For isolation of genomic DNA from whole blood, a commercially available kit (Wizard® Genomic DNA Purification Kit, Promega Corporation) was employed according to the manufacturer’s instruction.

For isolation of DNA from FACS-sorted leukocyte subsets, a commercially available Qiagen DNA Micro Kit was used according to the manufacturer’s instruction. Subsequently, DNA was amplified by Whole Genome Amplification using the commercially available Qiagen Repli-g kit.

### Capillary Sequencing

Capillary sequencing of genomic DNA from both patients was performed with primers designed for the variant in the *JAK3* gene with PrimerZ (www.primerz.org) and purchased from Eurofins/MWG Operon (Ebersberg, Germany). The sequences of the primers are AAGTGCTCTGACTTGCCACA (forward) and CACCTTTCTGACCCCTTCAC (reverse). Expand High Fidelity PCR System (Roche, Basel, Switzerland) was applied for PCR amplification and Big Dye Terminator v3.1 Cycle Sequencing Kit (Applied Biosystems, Darmstadt, Germany) for capillary sequencing. Sequences were acquired using an ABI 3130xl Sequencer (Applied Biosystems) and analyzed using 3130xl Genetic Analyzer (Applied Biosystems) and Sequencher DNA Software 4.10.1 (Gene Codes Corporation, Ann Arbor, MI, USA).

### Homozygosity Mapping

Homozygous intervals were determined as previously described [14] using Affymetrix® Genome-Wide Human SNP Array 6.0 technology. The outcome data was analyzed using Affymetrix® Genotyping Console^™^ software version 4.0.1.8.6. Homozygous intervals were mapped using Homozygosity Mapper (www.homozygositymapper.org/).

### Exome Sequencing and Data Analysis

Exome sequencing was performed for patient 2. Illumina TruSeq DNA Sample Preparation Guide and the Illumina TruSeq Exome Enrichment Guide version 3 were used. Genomic DNA (1 μg) was sheared to fragments of 200–300 bp. Blunt ending, adenylation and adapter-ligation allowing the fragments to hybridize onto the flow cell were carried out. Exonic DNA fragments were enriched and clusters were generated using the Illumina cBot Cluster Generation System following the TruSeq PE Cluster Kit v3 Reagent Preparation Guide. The DNA fragment clusters ran in a multiplexed pool with five other samples distributed on two lanes of the flow cell.

Data analysis was performed as previously described [14]. Reads were aligned using Burrows-Wheeler Aligner (BWA) to the human genome 19. Insertion/deletion realignment was performed as well as Genome Analysis Toolkit (GATK version 1.5)-based quality score recalibration. For single nucleotide variants (SNVs) and Deletion/Insertion variants (DIVs) calling, Unified Genotyper and GATK Variant quality score recalibration were performed. SNVs and DIVs lists were uploaded to SeattleSeq Annotation database with dbSNPbuild135. Variants present in 1000Genomes and dbSNP were excluded and the lists were filtered for nonsense, missense and splice-site variants present within the overlapping homozygous intervals of both patient. At last, SNVs were filtered according to a validation prediction score.

### Cell Sorting for Analysis of Somatic Chimerism

Peripheral blood mononuclear cells (PBMCs) of both patients were isolated by density gradient centrifugation using Ficoll-Hypaque (GE Healthcare, Uppsala, Sweden) and stained with the following antibodies: CD3-FITC, CD4-APC (BD, Biosciences, Schwechat, Austria), CD8-PECy7 (Beckmann Coulter, Krefeld, Germany), CD19-PerCPCy5.5 (eBioscience, Vienna, Austria) and CD56-V450 (BD, Biosciences, Austria). Subsequently, the stained cells were sorted into different subgroups of leukocytes CD3+CD4+CD8-T-cells, CD3+CD4-CD8+ T-cells and CD3-CD19+ B-cells using a MoFlo Astrios Cell Sorter from Beckmann Coulter.

### Chimerism Analysis for Maternal Cells

The screening for maternal cells in the peripheral blood of the patient investigated was performed by quantitative chimerism testing, as described previously [15, 16]. Five informative microsatellite/short tandem repeat (STR) markers including D3S3045, D4S2366, D12S1064, D16S539, D17S1290, and SE-33 were analyzed by PCR analysis and capillary electrophoresis with fluorescence-based detection [17].

### T-cell CDR3 Vβ Spectratyping

The examination of TCR Vβ repertoire was performed by spectratyping analysis as described by Pannetier et al. [18] with the modification of the sequence for primers listed in the table below.

**Table:** 

**Target of the primer**	**Sequence**
**Primers for variable regions**	
BV02	ACATACGAGCAAGGCGTCGA
BV04	CATCAGCCGCCCAAACCTAA
BV07	CAAGTCGCTTCTCACCTGAATGC
BV17	TGTGACATCGGCCCAAAAGAA
BV21	GGAGTAGACTCCACTCTAAG
BV24	CCCAGTTTGGAAAGCCAGTGACCC
**Primer for the constant region**	
CßB1 (used for BV05, BV06BC, BV20)	CGGGCTGCTCCTTGAGGGGCTGCG
**Fluorescently labeled primer for the constant region**	
FAM-marked primer for the constant region	ACACAGCGACCTCGGGTGGG

For the fragment analysis, sequences were acquired using an ABI 3130xl Sequencer (ABI Applied Biosystems) and analyzed using GeneMapper software version 3.7.

### Generation of an EBV-Transformed Cell Line

PBMCs were isolated from heparinized peripheral blood by density gradient centrifugation (Lymphoprep, Axis-Shield PoC AS, Oslo, Norway) and transformed with Epstein-Barr virus (EBV) using the supernatant from the B 95–8 marmoset cell line (ATCC, Rockville, MD) according to a standard protocol [19]. Growing cells were expanded in complete RPMI 1640 medium supplemented with 10 % heat-inactivated fetal calf serum (FCS, PAA), 2 mM L-glutamine, 100 IU/ml penicillin, and 100 μg/ml streptomycin (Gibco, Paisley, Scottland) at 37 °C in the presence of 5 % CO2.

### Analysis of JAK3 and STAT5 Protein Expression

EBV-transformed B-cells were lysed for 30 min in ice-cold lysis buffer [20 mM Tris–HCl (pH 7.5), 150 mM NaCl, 2 mM EDTA, 1 % Nonidet P-40, protease inhibitor cocktail (Complete; Roche)] and insoluble material was removed by centrifugation (16,000 x g, 10 min, 4 °C). 20 micrograms of protein was resolved by 8 % SDS-polyacrylamide gel electrophoresis (SDS-PAGE), electrotransferred onto polyvinylidene difluoride membrane (Immobilon-P; Millipore), and immunoblotted with anti-JAK3 antibody (C-21; Santa Cruz Biotechnology Inc., Santa Cruz, CA, USA), anti-Stat5 antibody (89; BD Biosciences) and anti-GAPDH antibody (Santa Cruz). Detection was performed using the SuperSignal West Pico ECL detection system (Thermo Scientific, Waltham, MA, USA).

### Analysis of JAK3 Signaling Function in B-cell Lines and CD4+ T-cells

Measurement of STAT3 and STAT5 activation was performed by flow cytometry: Phosphorylation-state analysis was performed on EBV-transformed B-cells or CD4+ T-cells contained within the PBMC fraction using BD PhosFlow technology, according to the manufacturer’s instructions (BD Biosciences, Mississauga, ON). After 2 h of culture in 1 % FCS, cells were stimulated with recombinant human IL-2 at 100, 1,000 and 10 000 U/ml (Bio-Rad AbD Serotec, Germany), IL-4 plus IL-21 at 100 ng/ml (Gibco/Life Technologies, Vienna, Austria) or IL-6 at 100 ng/ml (Bio-Rad AbD Serotec, Vienna, Austria) for 15 min at 37 °C, fixed with BD Cytofix Fixation buffer and permeabilized in ice-cold BD Perm buffer III (BD Biosciences). Cells were stained with Alexa Fluor 647-labeled anti-STAT3-pY705 or anti-STAT5-pY694 (both from BD Biosciences). CD4+ T-cells contained within the PBMC fraction were identified by gating in dual-colour flow cytometry. Fluorescence was measured by flow cytometry using a FACS Calibur (Becton Dickinson, Heidelberg, Germany) and data analyzed with the CellQuest software (Becton Dickinson).

### Crystal Structure

Crystal structures of the kinase domain of JAK3 tyrosine kinase were created using the ICM-Browser Software (Molsoft LLC, San Diego, US).

## Results

### Patient Characteristics

Patient 1 (currently 15 years of age) is the first child of healthy consanguineous parents of Turkish origin. She was admitted to hospital for the first time at the age of 20 months because of recurrent episodes of infection of the upper and lower airways with pulmonary infiltrates at varying locations, subsequently complicated by development of atelectases. Parenteral antibiotic therapy was initiated and led to improvement of clinical symptoms and discharge from hospital. No further severe infectious episodes occurred thereafter.

Because of the recurrent infectious episodes primary immunodeficiency was suspected and an immunological work-up was initiated which revealed CID with CD4 lymphopenia on multiple occasions, IgG2-IgG4-subclass deficiency, and selective antibody deficiency against bacterial polysaccharide antigens (Table 1 and online supplementary Table 2). Intravenous Immunoglobulin (IVIG) substitution therapy was initiated at the age of five years and continued for over a year. Re-evaluation of antibody production after cessation of IVIG therapy confirmed selective polysaccharide antibody deficiency and showed a decreased IgG antibody response against other antigens e.g. diphtheria toxoid vaccination (Table 1). At the age of nine, subcutaneous immunoglobulin (SCIG) home therapy was started which is still being carried out.

**Table 1:** Immunological phenotype of both patients at representative time points

A. Serum levels of immunoglobulins, antibacterial antibodies and antibodies against vaccination antigens								
	Patient 1 (II - 1)				Patient 2 (II - 2)			
	22 m		9 years		3 years		8 years	
IgG (mg/dl)	585	(570–1322)	743	(790–1700)	801	(696–1518)	943	(790–1700)
IgA (mg/dl)	114	(23–97)	144	(76–450)	48	(46–177)	43	(76–450)
IgM (mg/dl)	307	(76–187)	247	(90–350)	160	(97–228)	147	(90–350)
IgG1 (mg/dl)	497	(457–734)	554	(500–880)	616	(400–983)	732	(500–880)
IgG2 (mg/dl)	<23	(56–200)	21	(150–600)	60	(70–400)	108	(150–600)
IgG3 (mg/dl)	<6	(20–81)	22	(20–100)	24	(20–81)	36	(20–100)
IgG4 (mg/dl)	<8	(0–40)	<8	(8–120)	<6	(0–40)	<7	(8–120)
Tetanus-IgG (IU/ml)	0,45*^3^)	(> = 0,40)	1,57*^4^)	(> = 0,40)	0,6*^3^)	(> = 0,40)	1,02*^5^)	(> = 0,40)
Diphteria-IgG (IU/ml)	0,15*^3^)	(> = 0,40)	0,17*^4^)	(> = 0,40)	0,08*^3^)	(> = 0,40)	0,09*^5^)	(> = 0,40)
Pn23-IgG (reciprocal titer)	<20	(> = 200)	81*^1^)	(> = 200)	<20	(> = 200)	249*^2^)	(> = 200)
Pn23-IgM (reciprocal titer)	198	(> = 100)	393*^1^)	(> = 100)	414	(> = 100)	999*^2^)	(> = 100)
Hib-IgG (μg/ml)	5,41*^3^)	(> = 1)	0,17	(> = 1)	2,57*^3^)	(> = 1)	0,47	(> = 1)
B. Lymphocyte subpopulations								
	Patient 1 (II - 1)				Patient 2 (II - 2)			
	22 m		10 year		3 years		8 years	
CD4 (%Ly)	7	(31–66)	22	(31–66)	11	(31–66)	22	(31–66)
CD4 (abs.Nr/μl)	430	(386–2022)	326	(386–2022)	417	(386–2022)	554	(386–2022)
CD4 + CD45RA + (%Ly)	2	(11–38)	5	(11–38)	4	(11–38)	7	(11–38)
CD4 + CD45RA + (abs.Nr/μl)	95	(170–1097)	74	(170–1097)	152	(170–1097)	176	(170–1097)
CD8 (%Ly)	35	(7–41)	39	(21–43)	42	(7–41)	49	(21–43)
CD8 (abs.Nr/μl)	2150	(107–1175)	578	(297–1011)	1593	(107–1175)	1233	(297–1011)
CD8 + CD62L + CD45RA + (% of CD8+)	n.a.		10,7	(25–61)	n.a.		7,8	(25–61)
CD19 (%Ly)	15	(7–23)	7	(7–23)	18	(7–23)	13	(7–23)
CD19 (abs.Nr/μl)	921	(71–549)	104	(71–549)	683	(71–549)	327	(71–549)
CD56 (%Ly)	25	(6–29)	29	(6–29)	34	(6–29)	25	(6–29)
CD56 (abs.Nr/μl)	1536	(98–680)	430	(98–680)	1289	(98–680)	629	(98–680)
CD3 (%Ly)	10	(53–85)	54	(53–85)	30	(53–85)	62	(53–85)
CD3 (abs.Nr/μl)	614	(694–2976)	800	(694–2976)	1138	(694–2976)	1560	(694–2976)
HLA-DR (%Ly)	82	(4–18)	48	(10–36)	53	(10–36)	74	(10–36)
HLA-DR (abs.Nr/μl)	5036	(75–505)	711	(200–800)	2010	(200–800)	1862	(200–800)
CD3 + HLA-DR (%Ly)	7	(1–8)	23	(2–12)	13	(2–12)	44	(2–12)
CD3 + HLA-DR (abs.Nr/μl)	430	(19–219)	341	(20–250)	493	(20–250)	1107	(20–250)
C. Lymphoproliferative response to mitogenic stimulation (3H-thymidine incorporation)								
	Patient 1 (II - 1)				Patient 2 (II - 2)			
	22 m		10 year		3 years		8 years	
PHA 1.6μg (dpm)	10135	(> = 30000)	101821	(> = 20000)	25678	(> = 20000)	72068	(> = 20000)
CON A 1.2μg (dpm)	1415	(> = 5200)	69767	(> = 5000)	42126	(> = 5000)	63988	(> = 5000)
PWM 1:100 (dpm)	7450	(> = 40000)	45225	(> = 20000)	4599	(> = 20000)	47075	(> = 20000)
Medium (dpm)	183	(<=600)	60	(<=400)	75	(<=400)	109	(<=400)
D. B-cell subpopulations								
					Patient 1 (II - 1)		Patient 2 (II - 2)	
					15 years		11 year	
CD38+ CD24+ transitional B-cells (%CD19)					3,8	(3,9–7,8)	10,7	(3,9–7,8)
CD27-IgD+ naïve B-cells (%CD19)					76,3	(75,2–86,7)	70,1	(75,2–86,7)
CD27+ IgD+ non-switched memory B-cells (%CD19)					7,6	(4,6–10,2)	9,3	(4,6–10,2)
CD27+ IgD- switched memory B-cells (%CD19)					10,9	(3,3–9,6)	13,3	(3,3–9,6)
CD27-IgD- memory B-cells (%CD19)					5,2	(2,3–5,5)	7,2	(2,3–5,5)
CD24-CD38+ plasmablasts (%CD19)					2,2	(0,3–1,7)	1,3	(0,3–1,7)

Pn23, 23-valent pneumococcal polysaccharide vaccine; *HiB* Haemophilus influenzae Type B

%Ly, percentage of lymphocytes; abs.Nr/μl, absolute number/μl blood; n.a. = data not available

*PHA* phytohaemagglutinin; *CON* A Concanavalin A; *PWM* Pokeweed-Mitogen; dpm, disintegrations per minute

%CD19, percentage of CD19+ cells

Normal ranges are indicated in brackets, next to patient values

*^1^) measured following three vaccinations with Pn23 at the age of 5, 7 and 8 years

*^2^) measured following two vaccinations with Pn23 at the age of 3,5 and 7 years

*^3^) measured following four vaccinations

*^4^) measured 3 months after tetanus-diphtheria booster vaccination

*^5^) measured following a total of six vaccinations against tetanus and diphtheria

The siblings of patient 1 were tested for immunodeficiency due to positive family history. Only one sister (patient 2) showed CD4 lymphopenia on initial determination that improved during follow-up and IgG2-IgG4-subclass deficiency, while generation of selective polysaccharide antibodies was intact (Table 1, online supplementary Table 2). She received antibiotic prophylaxis until the age of six years. Immunological findings in the other two siblings were within normal range with the exception of a slight hypogammaglobulinemia but

intact antibody production against all antigens tested. Immunological findings in the parents were unremarkable (data not shown).

### Immunological Phenotype

#### T-Cell Deficiency

The immunological phenotype of both patients is depicted in Table 1. A marked reduction in the number of CD4+CD45RA+naïve T helper cells was observed in both patients, more pronounced in patient 1. Numbers of CD4+ memory T-cells were within the normal range while numbers of naïve CD8+ cells were also significantly reduced(CD8+CD62L+CD45RA+cells shown in Table 1). In contrast, numbers of naïve B lymphocytes were comparable to an age-matched control. CD4+ and CD3+ lymphocytes were below the normal range in both patients, while numbers of NK cells as identified by CD56 staining were increased initially but returned to normal during follow-up (Table 1 and online supplementary Fig 1). In patient 1, this predominant CD56+ lymphocyte cell type was further characterized and found to be positive for several markers including CD8, HLA-DR-dim, CD2, CD7, CD11b and negative for CD4 and CD3 (data not shown). Patient 1 initially showed a T-B+NK+SCID phenotype at the age of 22 months with low numbers of both CD4+ and CD8+ T-cells but with a normal number of total CD8 cells, most of which constituted NK-cells. During follow-up, this phenotype evolved into predominant CD4 lymphopenia. A comparable development from a combined CD4+ and CD8+ T-cell deficiency to predominant CD4 lymphopenia was also observed in patient 2 (Table 1 and supplementary Figure 1). The reduction in naïve CD4 cells is still detectable although it became less pronounced (Table 1 and online supplementary Fig 1).

T-cell activation in response to mitogenic stimuli was decreased in both patients during younger age and normalized during follow up (Table 1). Further investigation of the capacity of T-cells to respond to activation was performed in patient 1 and revealed a substantial impairment of TCR/CD3-dependent lymphoproliferative responses following stimulation with recall antigen (tetanus toxoid) despite four previous vaccinations; anti-CD3- and staphylococcal superantigen-induced T-cell proliferation was decreased as well (online supplementary table 1). Repeated booster vaccinations normalized the T-cell response to recall antigen, while mitogen-induced IL-2 and IFN-gamma release was still impaired (online supplementary table 1).

#### Evaluation of Antibody Production

In both patients, IgG2 and IgG4 subclass deficiency was present (Table 1). The IgG-response following four tetanus- and *haemophilus influenzae type B* vaccinations was within the normal range, and neutralizing antibodies against all three polio vaccine strains were detectable in patient 1 (serum titer of 1:1,280 against strains I to III). However, IgG antibody production against other vaccination antigens was impaired, diphtheria IgG antibodies remained low in both patients despite four vaccinations in the first two years of life and were only borderline detectable at the age of 10 years despite repeated booster vaccinations (Table 1). In patient 1, EBV capsid-IgG antibodies were positive and EBV–IgM antibodies negative, indicating a history of primary EBV infection; EBNA-IgG-antibodies remained negative up to the age of nine years (time of last follow-up control without SCIG substitution therapy). She had also experienced previous CMV infection as CMV-IgG antibodies were positive and CMV–IgM antibodies negative.

Patient 1 displayed a selective IgG antibody deficiency against bacterial polysaccharide antigens while in patient 2 this B-cell function was within the lower normal range (Online supplementary table 2). At the age of 2 to 3 years, pneumococcal IgG antibodies were undetectable in the serum of both patients who had never been vaccinated against pneumococcal polysaccharide before. Patient 1 failed to mount a significant IgG antibody response following each of three pneumococcal vaccinations at the age of 5, 7 and 8 years, while the IgM response was within the normal range. In contrast, IgG antibody production was within the lower normal range after two pneumococcal vaccinations in patient 2, and she developed a significant IgM response (online supplementary table 2).

### Molecular Genetic Analysis of the Underlying Defect

#### Analysis of Known Molecular Defects of T-Cell Deficiency

Several molecular defects known to lead to primary T-cell deficiency were examined in patient 1 and found to be normal, adenosine deaminase and purine nucleoside phosphorylase deficiency (examined by measuring the respective enzyme in peripheral blood erythrocytes), a defect in chromosome breakage repair (examined following treatment of cultured cells with Diepoxybutan), *NBS1*-gene deletion, and 22q11 microdeletion were excluded. Levels of alpha-1-fetoprotein in serum as a potential indication for ataxia telangiectasia as well as flow cytometric analysis of IL-7Rα, IL-2Rγ, CD3 und CD45 expression in peripheral blood leukocytes showed normal results.

#### Identification of a Mutation in *JAK3*

Given the family history including consanguinity, an autosomal recessive Mendelian trait was suspected. Thus, we employed a combination of homozygosity mapping and exome sequencing to detect the underlying genetic cause of the disease.

**Fig. 1:**
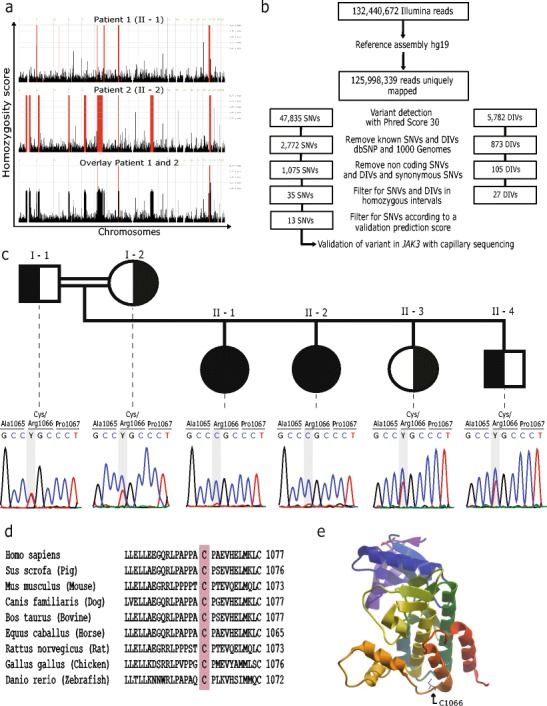
**a** Homozygosity mapping results showing homozygous intervals highlighted in red color **b** Filtering strategy for exome sequencing data **c** Pedigree of the index family with sequence of the *JAK3* mutation site highlighted in gray background **d** Multiple sequence alignment with the *JAK3* mutation site highlighted in red background **e** Crystal structure of the JAK3 kinase domain. The described mutation site is marked with a black arrow

Homozygosity mapping of the affected siblings revealed seven and thirteen homozygous intervals, respectively. Three intervals on chromosome six, eight and nineteen were homozygous in both patients (Fig 1a). As the causative genetic defect was assumed to be located in an overlapping homozygous interval, exome sequencing data were filtered for the described homozygous intervals (Fig 1b). Among those was a homozygous variant in the *JAK3* gene (c.T3196C, p.Cys1066Arg) encoding a tyrosine kinase bound to the common gamma chain of various interleukin receptors [20]. JAK3 deficiency is commonly known to cause SCID [21]. Using Sanger sequencing, this variant could be validated and it showed perfect segregation with the disease (Fig 1c). Multiple sequence alignment revealed that this position is highly conserved throughout evolution (Fig 1d) with a potentially critical function within the kinase domain of JAK3 (Fig 1e).

#### Vβ Spectratyping Indicates Restricted T-Cell Receptor (TCR) Repertoire

**Fig. 2:**
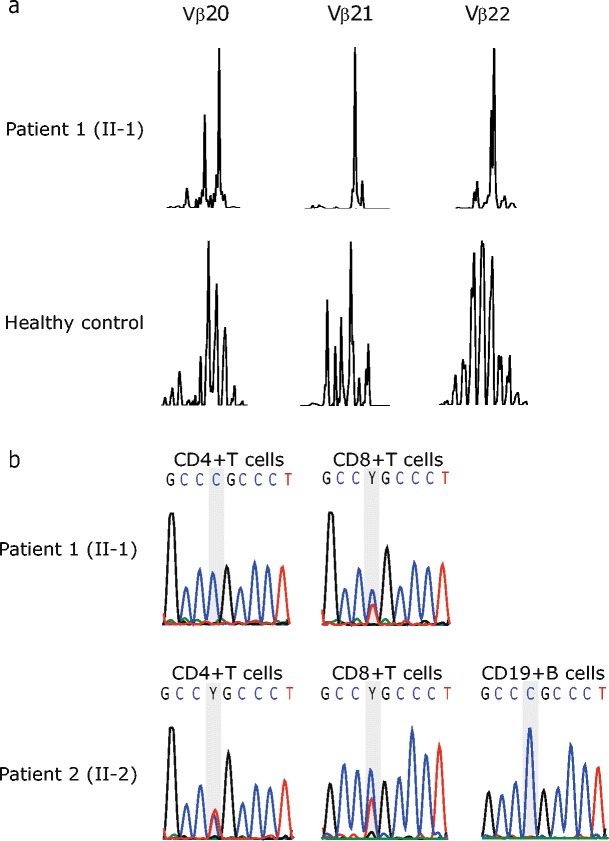
**a** Subfamilies Vβ20-22 of normal donor and patient 1 are shown as representatives of Vβ TCR Spectratyping data. **b** Sequencing of the *JAK3* in genomic DNA derived from sorted lymphocyte subsets of both index patients. The mutation site is highlighted with a gray background

TCR Vβ spectratyping is a well-established method to assess TCR diversity and restriction. Several T-cell deficiencies are known to use a restricted TCR Vβ repertoire [22, 23]. We employed TCR Vβ spectratyping to assess the clonality of the TCR repertoire and observed a restricted TCR repertoire in both index patients (Fig 2a and online supplementary Fig 2).

#### Detection of Somatic Chimerism

Somatic chimerism has been observed in several primary immunodeficiencies such as Wiskott Aldrich Syndrome [24–27], ADA-deficiency [28, 29] or X-linked SCID [30, 31] and is possibly associated with an amelioration of the clinical phenotype of disease [32]. Thus, we speculated whether somatic chimerism may have contributed to the relatively mild clinical phenotype despite the homozygous *JAK3* mutation in a critical protein domain in both index patients. Hence, CD4+ T-cells, CD8+ T-cells and CD19+ B-cells were FACS-sorted, followed by Sanger sequencing for an amplicon harboring the identified *JAK3* mutation. CD8+ T-cells of both index patients showed somatic chimerism whereas revertant CD4+ T-cells were present only in the second patient (Fig 2b). Whether this finding contributes to the milder clinical phenotype in this patient is unclear at the moment, as further studies are required to formally prove this assumption. To exclude maternal chimerism, DNA derived from total leukocyte preparations from peripheral blood was assessed and revealed no indication of maternal cells above the detection limit of 1 % (data not shown).

### Biological Relevance of the Observed *JAK3* Mutation for Cell Signaling Events

#### Analysis of JAK3 and STAT5 Protein Expression in B-cell Lines

**Fig. 3:**
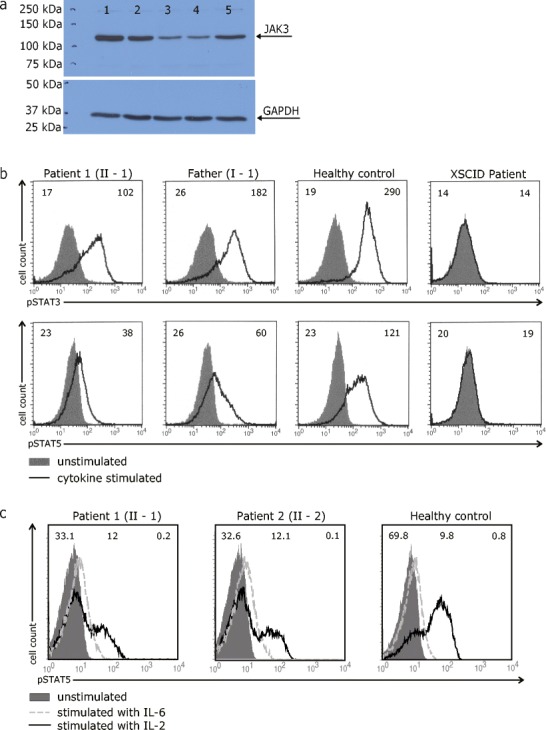
**a** Analysis of JAK3 protein expression in B-cell lines of a healthy control (1), father of the patients (2), patient 1 (3), patient 2 (4) and a γc-deficient SCID patient (5). Examination of GAPDH protein expression served as a loading control. **b** Analysis of JAK3 signaling function in B-cell lines of a healthy control, patient 1 (II-1), her father (I-1) and a γc-deficient SCID patient after stimulation with IL-4 and IL-21. Numbers in the top left and right corners of each histogram in panel B indicate the Mean Fluorescence Intensity (MFI) values of unstimulated and cytokine-stimulated cells, respectively. **c** Analysis of JAK3 signaling function in CD4+ peripheral blood T-cells of a healthy control, patient 1 (II-1) and patient 2 (II-2) after stimulation with IL-2. Histogram overlays represent intracellular levels of phosphorylated STAT5 in CD4+ T-cells without stimulation or after stimulation with IL-2 or IL-6. Numbers in the left top corner and middle part of each histogram indicate percentages of cells with a positive staining for pSTAT5 following stimulation with IL-2 or IL-6, respectively, while numbers in right corner constitute percentages of pSTAT5-positive unstimulated control cells

To further study possible biological consequences of the observed *JAK3* mutation on a cellular level, we generated EBV-transformed B-cell lines from the patients homozygous for the mutation, their heterozygous father and an unrelated healthy control. Sanger sequencing confirmed that the patients’ B-cell lines indeed showed a homozygous *JAK3* mutation, while heterozygous expression of this mutation was detected in the father’s B-cell line (online supplementary Fig 3). We then examined protein lysates from EBV-transformed B-cells to assess whether the mutation led to destabilization and consequently reduced protein levels of the altered JAK3. Western blot analysis showed presence of JAK3 protein in both patients at reduced levels compared to her father, the normal control or a γc-deficient XSCID patient (Fig 3a). STAT5 protein expression was comparable in all cell lines tested (data not shown).

#### Analysis of JAK3 Signaling Function in B-cell Lines and CD4+ T-Cells

IL-2 activates STAT5, IL-4 activates STAT5 and STAT6 [33], while IL-21 activates STAT1, STAT3 and STAT5 in a JAK3-dependent manner [34]. We exposed EBV-transformed B-cells from patient 1 and her father to IL-4 and IL-21 and assessed activation of STAT3 and STAT5 by flow cytometry with phospho-STAT (p-STAT) specific antibodies. Results show that STAT3 phosphorylation was clearly detectable but diminished to a different extent in patient 1 and her father as compared to the healthy control (Fig 3b). Furthermore, STAT5 phosphorylation was severely decreased in patient 1 while being moderately reduced in her father. In a second run, B-cells of patient 1 and 2 were stimulated with IL-2 in order to quantify phosphorylation of STAT5 (online supplementary Fig 4). Compared to the healthy control, it was remarkably diminished but still detectable. Both experiments showed that neither STAT3 nor STAT5 phosphorylation were activated in a γc-deficient XSCID B-cell line. We next investigated JAK3-signaling in CD4+ T-cells from the two patients and a healthy control. PBMCs were stimulated with IL-2 before activation of STAT5 was assessed by dual colour flow cytometry and gating on CD4+ lymphocytes. We observed that the presence of revertant CD4+ T-cells in patient 2 had no effect on IL-2-induced, JAK3-dependent STAT5-phosphorylation which was diminished to a comparable extent in both patients (Fig. 3c). Since IL-6 is known to induce both STAT3 phosphorylation [35] and, to a lesser extent, STAT5 phosphorylation [36], in a JAK3-independent manner, we investigated intactness of these pathways in PBMCs from the patients under study. IL-6-induced STAT5-phosphorylation (shown in Fig. 3c) as well as STAT3-phosphorylation (29,8 % in healthy control and 29,8 % in patient 2, data not illustrated) were both normal. This confirms the presence of a JAK3-specific signaling defect in the patients’ CD4+ T-cells.

## Discussion

JAK3 is an intracellular protein tyrosine kinase which is predominantly expressed in hematopoietic cells and belongs to the Janus kinase family. It binds to the common gamma chain (γc) of receptors of interleukin(IL)-2, IL-4, IL-7, IL-9, IL-15 and IL-21 and plays an essential role in cytokine receptor signaling pathway [37]. Upon ligand binding of the IL receptors, JAK3 is activated by autophosphorylation, enabling binding and phosphorylation of different STAT proteins including STAT1, 3, 5 and 6 [38, 39]. Consequently, STATs can build homodimers (and heterodimers [40]) and translocate to the cell nucleus in order to regulate the expression of several genes involved in development, proliferation and function of lymphoid cells [41].

Similar to γc chain-deficient X-linked SCID, deficiency of JAK3 is known to cause autosomal recessive SCID with a reduced number of T-cells and natural killer cells as well as dysfunctional B-cells in normal cell count (T^−^B^+^NK^−^ SCID) and hypogammaglobulinemia [42]. As in other SCID subtypes, affected children typically suffer from recurrent or persistent infections, often with opportunistic pathogens, intractable diarrhea, thrush and failure to thrive. Without prompt and adequate therapy by means of stem cell transplantation, they have a significant mortality during the first two years of life [41, 43, 44]. Recently, hypomorphic mutations in *JAK3* have been reported to widen the spectrum of clinical and immunological phenotypes of JAK3 deficiency to include T-cell lymphopenia with maternal T-cell engraftment and defective antibody responses [45].

Due to the severe phenotype associated with deficiency of JAK3, it was surprising to find a mutation in *JAK3* in the index family with a relatively mild phenotype in terms of CID evolving into predominant CD4 deficiency. In retrospect, patient 1 showed a T-B+NK+SCID at the age of 22 months with a low number of CD3 lymphocytes, low numbers of CD4+ and CD8+ T-cells but with a normal number of total CD8 cells, most of which constituted NK-cells. During follow-up, this phenotype evolved into predominant CD4 lymphopenia. A comparable development from (S)CID to predominant CD4 lymphopenia was also observed in patient 2 (Table 1 and supplementary Fig. 1).

The described novel homozygous mutation in *JAK3* (c.T3196C, p.Cys1066Arg) affects a highly conserved amino acid site which is located in the kinase domain of JAK3 [46]. Similar to other JAK3 deficient patients [22, 23], we observed an oligoclonal restriction of the TCR Vβ repertoire in both index patients (Fig 2a and online supplementary Fig 2).

To investigate the molecular etiology of the relatively mild clinical disease phenotype in both index patients, we hypothesized that (i) the novel missense mutation may be of hypomorphic nature or (ii) a molecular chimerism event may have occurred in these patients.

In other primary immunodeficiency disorders, such molecular chimerism has been linked to atypical and relatively milder disease courses, including ADA-deficient SCID [28, 29], X-linked ectodermal dysplasia and immunodeficiency [47], γc deficiency [30, 31] or Wiskott-Aldrich syndrome [24–27]. Our results from Sanger sequencing of DNA extracted from FACS-sorted leukocyte subsets indeed revealed somatic chimerism in CD8+ T-cells in both patients and additional chimerism in CD4+ T-cells in patient 2 (Fig 2b). In view of this constellation, it is interesting to note that patient 1 with no somatic reversion in CD4+ T-cells presented in childhood with recurrent infections of the respiratory tract and received IVIG substitution therapy whereas patient 2 with somatic chimerism in CD4+ T-cells showed no clinical sign of immunodeficiency and merely laboratory investigations showed CD4+ lymphocytopenia and IgG subclass deficiency. The presence of somatic reversion in both patients of the same family despite somatic reversion being a rare event indicates a selective proliferative advantage of lymphocytes with a reverted over non-reverted *JAK3* genotype due to restored development and differentiation. Although it is tempting to assume that the somatic chimerism may have attenuated the severity of the disease phenotype in the described individuals, formal proof of this assumption would clearly require further studies. Preliminary results show that the presence of revertant cells had no effect on the level of residual JAK3 signaling activity when CD4+ T-cells from both patients were compared. Furthermore, the present data do not allow for exact quantification of the percentage of revertant cells contained within the T-cell subsets.

Interestingly, analyses of phosphorylation of STAT3 and STAT5 in an EBV-transformed B-cell line from patient 1 and from the heterozygous father after stimulation with IL-4 and IL-21 showed detectable levels of phosphorylated STATs that were diminished to a different extent. Although reduced JAK3 protein expression might have contributed to this finding, it suggests that the specific *JAK3* mutation in this family may be hypomorphic, thus allowing for residual γc- and JAK3-mediated signaling function, which is a likely explanation for the relatively mild clinical phenotype observed in our JAK3-deficient patients. The phenomenon of null or hypomorphic mutations in the same gene causing different phenotypes of immunodeficiencies has already been described previously. For instance, null mutations of *RAG1* are known to cause SCID while hypomorphic mutations have been associated with Omenn syndrome [48] or idiopathic CD4 lymphopenia [13].

## Conclusions

Taken together, we here describe for the first time JAK3 deficiency due to a hypomorphic *JAK3* mutation and with somatic chimerism, causing a phenotype of T-cell deficiency evolving into predominant CD4 lymphopenia. It is conceivable that other patients with primary CD4 lymphopenia may, in a similar fashion, bear hypomorphic mutations and/or somatic chimerism in other genes which are usually associated with SCID phenotypes. However, it appears likely that other subgroups of patients with CD4 lymphopenia are caused by novel nosological entities involved in T-cell homeostasis. Due to improvement of genomic technologies, the number of gene defects causing an incomplete impairment of T-cell development is increasing, leading to a broader understanding of normal and pathologic immune system development [49]. The availability of state-of-the art genomic technologies such as so-called “next generation sequencing” approaches will be instrumental in defining and classifying the range of genomic variations underlying this group of immunodeficiencies.

## Electronic supplementary material

Below is the link to the electronic supplementary material.

ESM 1 (PDF 123 kb)

ESM 2 (PDF 118 kb)

ESM 3 (PDF 243 kb)

ESM 4 (PDF 582 kb)

ESM 5 (PDF 253 kb)

ESM 6 (PDF 299 kb)
