# Supplementary material for: Combined Immunodeficiency Evolving into Predominant CD4+ Lymphopenia Caused by Somatic Chimerism in JAK3
Source: J Clin Immunol. 2014 Sep 10;34(8):941–53. doi: 10.1007/s10875-014-0088-2 (PMC4220108; doi:10.1007/s10875-014-0088-2)
Supplement: Supplementary file 1 — (PDF 123 kb) [file 10875_2014_88_MOESM1_ESM.pdf]

Supplementary Table 1: Impaired T cell activation in response to stimulation with mitogens, anti-CD3, superantigens and recall antigen in patient 1 (II-1)

A. Lymphoproliferative response (in dpm, 3H-thymidine incorporation)

	healthy control	patient 1 (23m)
PHA (1:1250)	27210	4109
ConA (12 µg/ml)	1240	5510
PWM (1:1000)	35999	2468
anti-CD3/OKT3 (10ng/ml)	11140	97
SE-A (50ng/ml)	71224	7086
SE-B (50ng/ml)	68166	7937
SE-E (50ng/ml)	78513	1800
TSST-1 (50ng/ml)	33724	16091
TTs (10LF/ml)	24507	73*[1])

	healthy control	patient 1 (5y1m)
Medium	429	91
PWM (1:1000)	15777	12963
TTs (4LF/ml)	11516	6372*[2])

*[1]) patient 1 has been vaccinated four times with tetanus toxoid

*[2]) measured following two booster vaccinations with tetanus toxoid

B. Interleukin-2 release (in pg/ml, ELISA, following 48hr-stimulation of PBMC)

	healthy control	patient 1 (5y1m)
Medium	65	86
PHA (1:1250)	5385	186

C. Interferon-gamma release (in pg/ml, ELISA, following 72hr-stimulation of PBMC)

	healthy control	patient 1 (5y1m)
Medium	21	<11
PHA (1:1250)	62981	410

dpm, disintegrations per minute; PHA, Phytohaemagglutinin; ConA, Concanavalin A; PWM, Pokeweed-Mitogen; SE, staphylococcal enterotoxin; TSST, toxic shock syndrome toxin; TTs, soluble tetanus toxoid; LF, LF-units (limit of flocculation units)
