# Supplementary material for: Combined Immunodeficiency Evolving into Predominant CD4+ Lymphopenia Caused by Somatic Chimerism in JAK3
Source: J Clin Immunol. 2014 Sep 10;34(8):941–53. doi: 10.1007/s10875-014-0088-2 (PMC4220108; doi:10.1007/s10875-014-0088-2)
Supplement: Supplementary file 2 — (PDF 118 kb) [file 10875_2014_88_MOESM2_ESM.pdf]

Supplementary Table 2: Antibody response following vaccination with bacterial polysaccharide antigen

Patient 1 (II - 1)

Age	20m	26m	4y 9m	4y 11m		5y 1m		7y 8m		8y	9y	9y 3m
Pn23-IgG *[1])	<20	<20	64	64	Vac*[3])	33	Vac*[3])	54	Vac*[3])	81	47	41
Pn23-IgM *[2])	198	185	357	384		347		609		393	459	860

Patient 2 (II - 2)

Age		3y 2m		3y 6m	5y	6y		7y 5m	8y 2m	9y 2m	10y 2m	11y 2m
Pn23-IgG *[1])		<20	Vac*[3])	399	105	37	Vac*[3])	316	249	120	185	159
Pn23-IgM *[2])		414		1129	508	340		636	999	365	376	413

values represent reciprocal serum titer of IgG and IgM antibodies determined by ELISA

*[1]) normal range in healthy Pn23-vaccinated controls is >=200

*[2]) normal range in healthy Pn23-vaccinated controls is >=100

*[3]) Vaccination against Pn23

Pn23, 23-valent pneumococcal polysaccharide vaccine; y, years; m, months
