# Supplementary material for: Combined Immunodeficiency Evolving into Predominant CD4+ Lymphopenia Caused by Somatic Chimerism in JAK3
Source: J Clin Immunol. 2014 Sep 10;34(8):941–53. doi: 10.1007/s10875-014-0088-2 (PMC4220108; doi:10.1007/s10875-014-0088-2)
Supplement: Supplementary file 3 — (PDF 243 kb) [file 10875_2014_88_MOESM3_ESM.pdf]

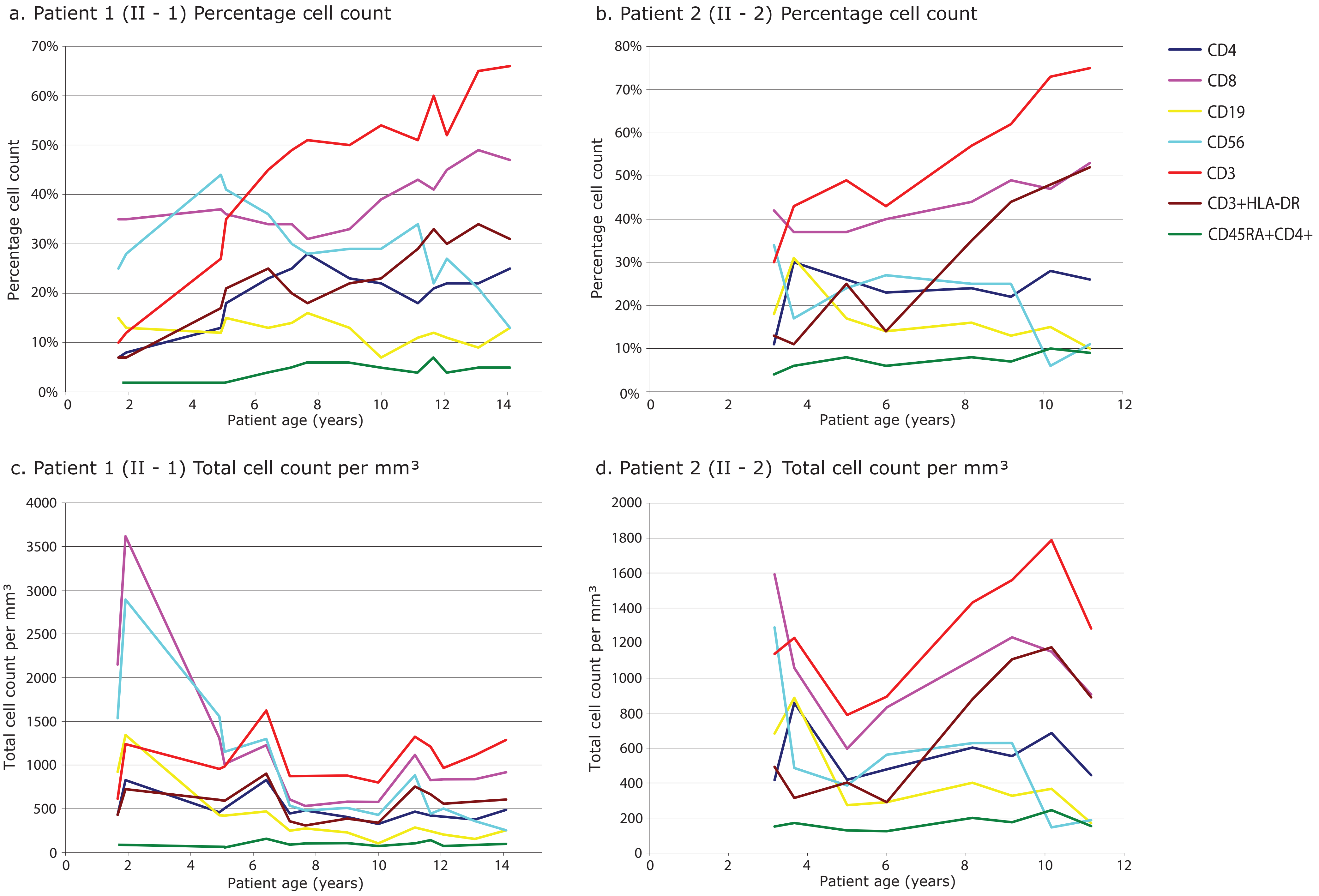

Supplementary Fig 1 Longitudinal follow-up of the immunological phenotype in both patients
