# Supplementary material for: Combined Immunodeficiency Evolving into Predominant CD4+ Lymphopenia Caused by Somatic Chimerism in JAK3
Source: J Clin Immunol. 2014 Sep 10;34(8):941–53. doi: 10.1007/s10875-014-0088-2 (PMC4220108; doi:10.1007/s10875-014-0088-2)
Supplement: Supplementary file 4 — (PDF 582 kb) [file 10875_2014_88_MOESM4_ESM.pdf]

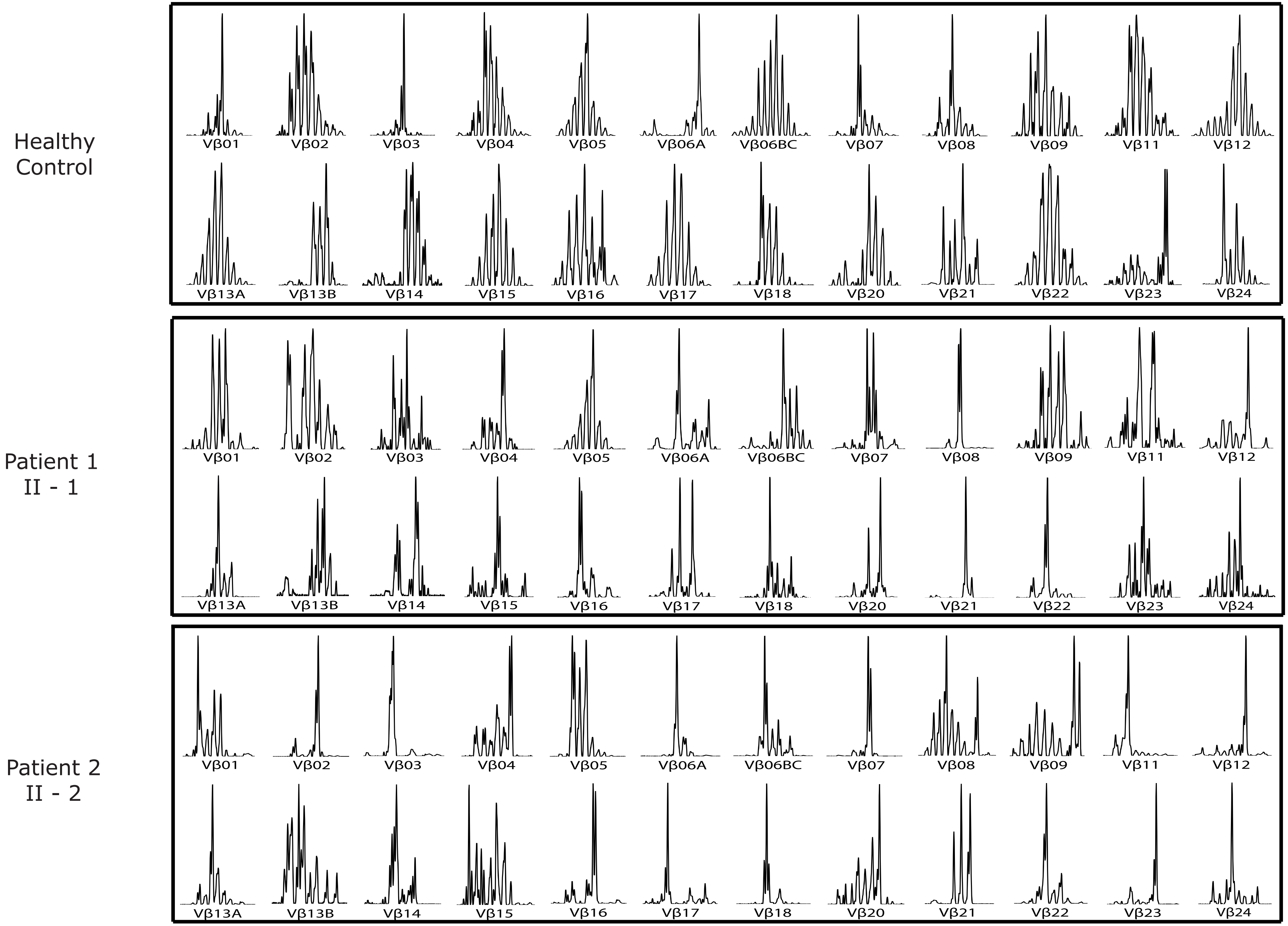

Supplementary Fig 2 Vβ TCR spectratyping of patient 1 (II-1), patient 2 (II-2) and a healthy control
