# Supplementary material for: Combined Immunodeficiency Evolving into Predominant CD4+ Lymphopenia Caused by Somatic Chimerism in JAK3
Source: J Clin Immunol. 2014 Sep 10;34(8):941–53. doi: 10.1007/s10875-014-0088-2 (PMC4220108; doi:10.1007/s10875-014-0088-2)
Supplement: Supplementary file 5 — (PDF 253 kb) [file 10875_2014_88_MOESM5_ESM.pdf]

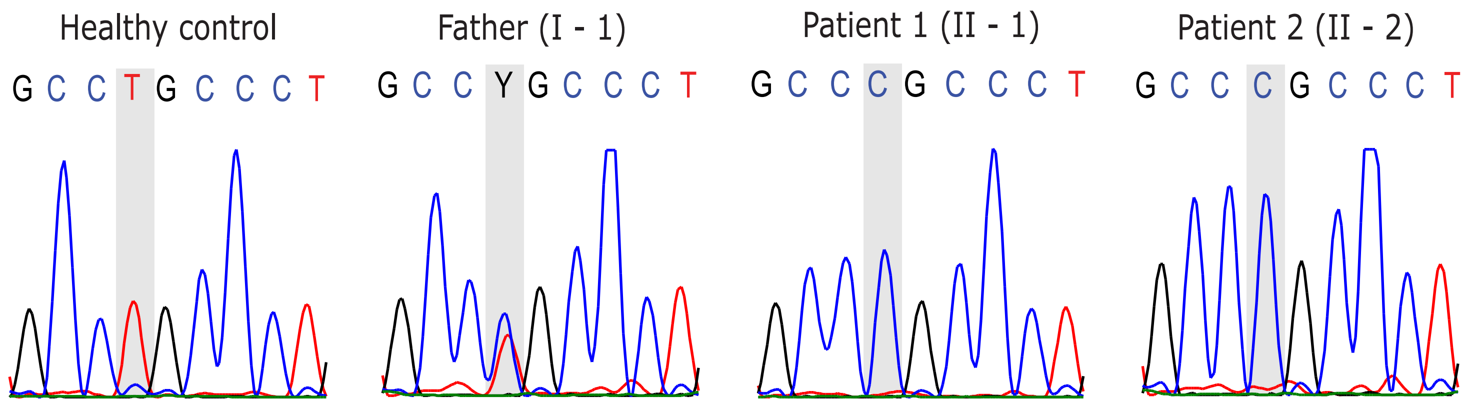

Supplementary Fig 3 Sequencing of exon 23 of the JAK 3 gene in genomic DNA derived from EBV- transformed B-cells from both index patients (II-1, II-2), their father and a healthy control. The mutation site of interest is highlighted in gray background.
