# Supplementary material for: Combined Immunodeficiency Evolving into Predominant CD4+ Lymphopenia Caused by Somatic Chimerism in JAK3
Source: J Clin Immunol. 2014 Sep 10;34(8):941–53. doi: 10.1007/s10875-014-0088-2 (PMC4220108; doi:10.1007/s10875-014-0088-2)
Supplement: Supplementary file 6 — (PDF 299 kb) [file 10875_2014_88_MOESM6_ESM.pdf]

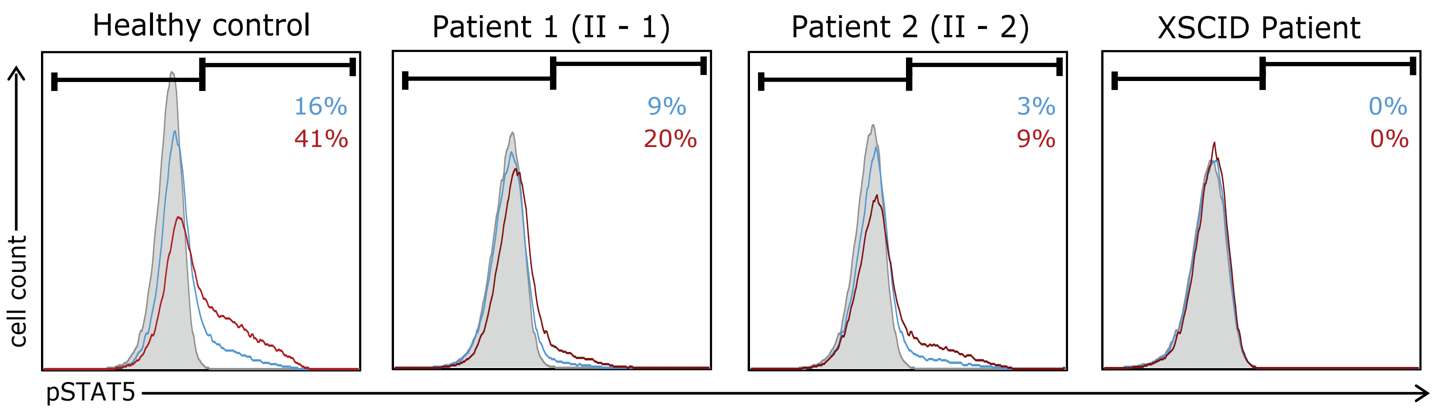

Supplementary Fig 4 Analysis of JAK3 signaling function in B cell lines of a healthy control, patient 1 (II-1), patient 2 (II-2) and a γc-deficient SCID (XSCID) patient after stimulation with IL-2. Histogram overlays represent intracellular levels of phosphorylated STAT5 in B cell lines without stimulation (gray line) or after stimulation with IL-2 at 1000 U/ml (blue line) and at 10 000 U/ml (red line). The percentages of cells that were stained phospho-STAT5-positive are indicated.
